# Multiple primary lung cancer displaying different EGFR and PTEN molecular profiles

**DOI:** 10.18632/oncotarget.13046

**Published:** 2016-11-03

**Authors:** Zhouyu Zhu, Tao Yu, Ying Chai

**Affiliations:** ^1^ Department of Thoracic Surgery, The Second Affiliated Hospital, College of Medicine, Zhejiang University, Hangzhou, China

**Keywords:** multiple primary lung cancer, EGFR, PTEN, gefitinib

## Abstract

We present a case of multiple primary lung cancer(MPLC) displaying heterogeneous EGFR and PTEN molecular profiles. Considering the physical condition of patients, the patient underwent surgical resection of the right lung lesion displaying gefitinib-insensitive and continued gefitinib treatment for the gefitinib-sensitive lesions in the left lung. As far as we know, this is the first report documenting a case of an integrated therapy strategy using surgical resection with gefitinib treatment for MPLC displaying different EGFR and PTEN molecular profiles. The patient has been in progression free survival up to now. Our experience could provide a treatment strategy for patients with similar disease.

## INTRODUCTION

Due to the availability of high-resolution thoracic imaging techniques, the number of patients dragonized as MPLC increases. However, distinguishing synchronous primary lesions from metastases was not always easily and mutation analysis sometimes was used to provide an accurate diagnosis. [1] To our knowledge, the therapeutic method for MPLC patients with heterogeneous EGFR molecular profiles and different response to EGFR-TKIs is not definite. Here we present a novel treatment strategy to a case of MPLC displaying heterogeneous EGFR and PTEN molecular profiles.

## CASE REPORT

**Figure 1:**
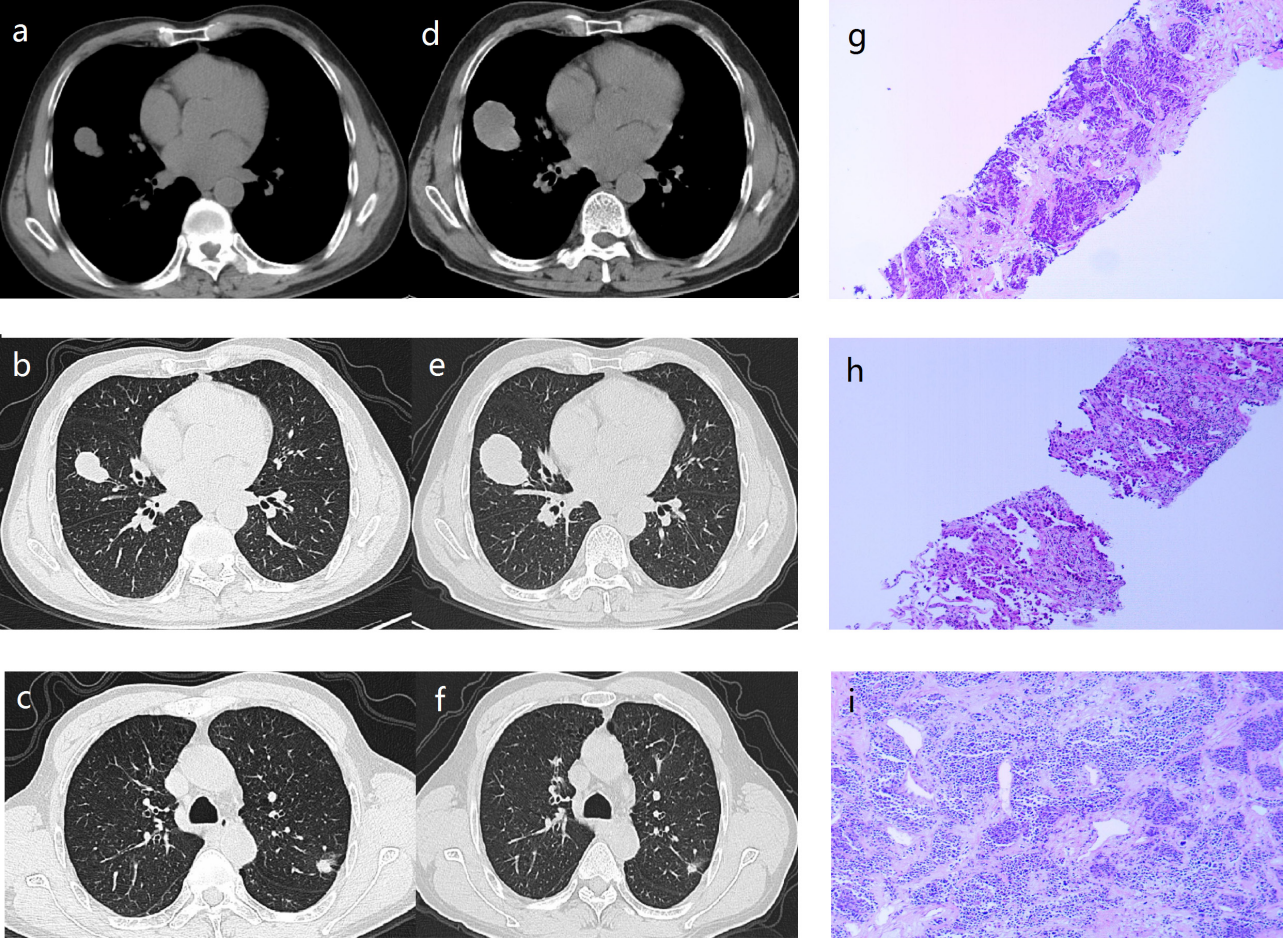
a.,b.,c. Computed tomographic scan obtained January 2016, showing a mass in the middle lobe of right lung measuring 3.0 x 2.0 cm, a nodule in the superior lobe of left lung measuring 1.4 x 0.9 cm. **d.**,**e.**,**f.** Computed tomographic scan obtained March 2016, showing the right middle lobe lesion had increased in size, whereas the left lung nodules had gotten smaller (compared with the computed tomographic scan obtained January 2016). **g.** Histopathologic examination of the right middle lobe lesion showed poorly differentiated carcinoma (hematoxylin and eosin; ×100). **h.** Histopathologic examination of the left upper lobe lesion showed adenocarcinoma (hematoxylin and eosin; ×100). **i.** Histopathologic examination of the right middle lobe showed a 4.0 x 3.5 cm poorly differentiated carcinoma (hematoxylin and eosin; ×100)

In January 2016, a 62-year-old asymptomatic male smoker was admitted to our hospital after being found bilateral lung masses. The PET-CT examination in the First Affiliated Hospital of Medical College of Zhejiang University revealed multiple nodules in bilateral lung with increased FDG metabolic abnormalities as the biggest in the middle lobe of right lung and the bilateral hilar lymph nodes enlargement. No distant metastases were detected. The patient denied any other medical history. On hospital admission, physical examination and laboratory test results were normal. A chest computed tomographic(CT) scan revealed a mass in the middle lobe of right lung measuring 3.0 x 2.0 cm, a nodule in the superior lobe of left lung measuring 1.4 x 0.9 cm. [Figure 1a, 1b and 1c] The possibility of MPLC or a primary lung cancer with multiple intrapulmonary metastases was considered. After obtaining the patient's informed consent, computed tomography (CT)-guided percutaneous transthoracic biopsy of the left upper lobe (LUL) lesion and the right middle lobe (RML) lesion was performed, and histopathologic examination indicated poorly differentiated carcinoma in the RML lesion [Figure 1g] and adenocarcinoma in the LUL lesion [Figure 1h]. In light of the patient's advanced age with bilateral multiple lung nodules, aggressive surgical treatment of the bilateral lesions was not feasible. The patient refused to undergo chemotherapy or radiotherapy. Mutation analysis was performed showed that tumor cells in the LUL lesion harbored mutation within exon 21 (L858R) of the EGFR gene with no KRAS or PTEN gene mutation was identified. Considering that the left upper lung cancer was positive for EGFR mutations, the patient began treatment of 250 mg gefitinib daily from January 2016. However, after 2 months of therapy, chest CT showed that the RML lesion had increased in size, whereas the left lung nodules had gotten smaller. [Figure 1d, 1e and 1f] Considering the opposite responses to gefitinib, our medical team chose a strategy of resection of the RML lesion and continued gefitinib treatment for other lesions. After obtaining the patient's informed consent, a radical resection of right middle pulmonary carcinoma and mediastinal lymph node sampling by video-assisted thoracic surgery was performed. Histopathologic examination showed a 4.0 x 3.5 cm poorly differentiated carcinoma of the right middle lobe. [Figure 1i] All resected lymph nodes were negative. Mutation analysis of the resected lesion was performed showed that the RML lesion carried a PTEN mutation, whereas no EGFR or KRAS mutation was detected. Thus we diagnosed the present case as synchronous primary lung cancer. The patient made an uneventful recovery and gefitinib treatment was continued. The patient has been in progression free survival up to now.

## DISCUSSION

According to generally accepted criteria for the diagnosis of MPLC, which had been originally proposed by Martini and Melamed, [2] the incidence of MPLC has been reported to be between 3.9% and 5.8%. [3-4] Distinguishing MPLC from pulmonary metastasis is difficult, especially the same histologic cell type identified. In such situation, mutation analysis may contribute to obtaining an accurate diagnosis. In our case, the patient's LUL and RML lesions exhibited different EGFR and PTEN molecular profiles, which were consistent with their histologic characteristics and the diagnosis of MPLC.

If conditions permit, the most effective treatment option for the patient is surgical treatment. However, the patient's physical condition was not suitable for surgery and initially treated with gefitinib has been taken to him. In light to the PTEN loss contributing to EGFR-TKIs resistance in NCSLC patient [5, 6] and the lesions' different responses to gefitinib, we chose a strategy of surgical resection of the right lung lesion displaying gefitinib-insensitive and continued gefitinib treatment for the gefitinib-sensitive lesions in the left lung. As far as we know, this is the first report documenting a case of an integrated therapy strategy using surgical resection with gefitinib treatment for MPLC displaying different EGFR and PTEN molecular profiles.

In conclusion, we presented a case of MPLC displaying heterogeneous EGFR and PTEN molecular profiles and provided a strategy for the patient. Doctors could consider this treatment strategy for patients with similar disease. To date, the patient is still alive, receiving gefitinib treatment, with no disease progression.
