# Addendum: Multiple primary lung cancer displaying different EGFR and PTEN molecular profiles

**DOI:** 10.18632/oncotarget.28313

**Published:** 2022-11-17

**Authors:** Zhouyu Zhu, Tao Yu, Ying Chai

**Affiliations:** ^1^Department of Thoracic Surgery, The Second Affiliated Hospital, College of Medicine, Zhejiang University, Hangzhou, China

**This article has an addendum:** Patient consent was obtained from the patient.

Original article: Oncotarget. 2016; 7:81969–81971. 81969-81971. https://doi.org/10.18632/oncotarget.13046

